# Comparative analyses of *Stvb*-allelic genes reveal *japonica* specificity of rice stripe resistance in *Oryza sativa*

**DOI:** 10.1270/jsbbs.22027

**Published:** 2022-12-06

**Authors:** Keiko Hayashi, Yoshihiro Kawahara, Hideo Maeda, Yuriko Hayano-Saito

**Affiliations:** 1 Institute of Agrobiological Science, NARO, Tsukuba, Ibaraki 305-8604, Japan; 2 Institute of Crop Science, NARO, Tsukuba, Ibaraki 305-8518, Japan

**Keywords:** rice stripe, resistance gene, genetic structure, functional marker, evolution, *Oryza* species

## Abstract

Rice stripe, a viral disease, causes widespread damage to *japonica* rice (*Oryza sativa* ssp. *japonica*). A rice stripe virus (RSV) bioassay revealed that many *indica* and *japonica* upland varieties exhibit resistance, whereas *japonica* paddy varieties are susceptible. However, the genetic background for this subspecies-dependent resistance is unclear. Herein, we focused on rice stripe resistance genes located at the *Stvb* locus. Three resistant alleles, *Stvb-i* (*indica*), *Stvb* (*japonica* upland), and *Stvb-o* (*Oryza officinalis*) were compared with the susceptible allele, *stvb-j* (*japonica* paddy). The expression of the resistance genes was higher than that of *stvb-j*. Sequence comparison revealed that the resistant and susceptible alleles had different 5ʹ-end sequences and 61-bp element(s) in the fourth intron. The insertion of an LTR-retrotransposon modified the exon 1 sequence of *stvb-j*. We then developed four DNA markers based on gene structure information and genotyped resistant and susceptible varieties. The LTR-retrotransposon insertion was detected only in susceptible varieties. Resistant genotypes were primarily found in *indica* and upland *japonica*, whereas paddy *japonica* carried the susceptible genotype. Our results characterize the genetic differences associated with RSV resistance and susceptibility in *O. sativa* and provide insights on the application of DNA markers in rice stripe disease management.

## Introduction

Rice stripe, caused by the vector-mediated rice stripe virus (RSV), impacts rice production. It is widely distributed across the major rice-growing areas of the world inhabited by small brown planthopper (*Laodelphax striatellus* (Fallén, 1826)). In Japan, RSV outbreaks were reported in the mid-twentieth century (Hibino 1996, Washio *et al.* 1968c) causing damage to more than 500,000 ha of rice fields per year, from 1963 to 1968 (Shinkai 1985). Since these outbreaks, research has focused on identifying rice stripe resistance genes to facilitate marker-assisted selection in breeding programs.

Rice stripe resistance genes, *Stva* and *Stvb*, were first identified in upland *japonica* rice varieties (Washio *et al.* 1968a, 1968c) and mapped to chromosomes 2 and 11, respectively (Maeda *et al.* 2006). Genetic analyses revealed that *Stvb* is a component of the multi-allelic locus and is thought to play a significant role in RSV resistance (Washio *et al.* 1968b, 1968c). Five *indica* rice genes, including *Stvb-i*, *STV11^IR24^*, *STV11^TQ^*, *STV11^KAS^*, and *STV11^SG^*, as well as one wild rice gene, were identified at the *Stvb* locus and mapped to the long arm of chromosome 11 (Hayano-Saito *et al.* 2000, Kwon *et al.* 2012, Maeda *et al.* 2007, Wang *et al.* 2011, Wu *et al.* 2011, Zhang *et al.* 2011). *Stvb-i*, which was introgressed into *japonica* varieties from the *indica* rice variety ‘Modan’ (Hayano-Saito *et al.* 1998), has maintained resistance to RSV for over half a century in Japan (Hayano-Saito 2014).

We predicted that *Stvb-i* encodes a 1,649 amino acid long ATP-binding domain protein belonging to the histidine kinase/HSP90-like ATPase superfamily (IPR036890) in the variety ‘St No. 1’ developed from ‘Modan’ (Hayano-Saito and Hayashi 2020). RSV can coexist with *Stvb-i* harboring plants (Hayano-Saito and Hayashi 2020); consequently, *Stvb-i-*mediated resistance exerts low pressure on pathogen survival and is associated with a lower risk of resistance breakdown (Brown 2015). In response to RSV inoculation, *Stvb-i* expression is particularly high in the meristematic tissues which suppresses the viral RNA multiplication in the ‘St No. 1’ variety. On the contrary, *stvb-j* (*Os11g0514000*/LOC_*Os11g31480*) exhibits low expression in the meristematic tissues of the susceptible *japonica* variety ‘Nipponbare’. This implies that *Stvb-i* expression in meristematic tissues may play a critical role in conferring RSV resistance (Hayano-Saito and Hayashi 2020).

Interestingly, RSV-affected regions remain limited to temperate East Asia, especially China, Korea, and Japan, which are major areas of *japonica* rice cultivation (Abo and Sy 1998, Cho *et al.* 2013, He *et al.* 2016, Xu *et al.* 2021). RSV specifically reduces the production of *japonica* variety (*Oryza sativa* L. ssp. *japonica*; Abo and Sy 1998, Cho *et al.* 2013), which significantly affects rice production, particularly in these countries (Hibino 1996, Shiba *et al.* 2018, Wu *et al.* 2009). In fact, in the 2000s, RSV caused a 30–40% reduction in rice production across eastern China (Wu *et al.* 2009). Several reports have indicated that this reduction was caused by the RSV-susceptibility of *japonica* rice (He *et al.* 2016, Otuka 2013, Yu *et al.* 2014). Among the *japonica* rice varieties, paddy varieties (grown in standing water in paddy fields) are more susceptible to RSV, whereas most of the upland varieties (grown in dry fields) are resistant (Washio *et al.* 1967, 1968c). Conversely, rice stripe has not been reported in any of the Asian countries where *indica* rice (*O. sativa* L. ssp. *indica*), which is RSV-resistant (Wang *et al.* 2014, Washio *et al.* 1967, 1968b, 1968c, Wu *et al.* 2009, Yu *et al.* 2014), is primarily cultivated. Therefore, RSV appears to be highly infectious to *O. sativa* varieties, depending on the subspecies.

In the present study, we attempted to identify genetic differences among *Stvb*-alleles to understand the subspecies dependency of *O. sativa* on RSV resistance. More specifically, we compared the gene sequences of three resistant alleles at the *Stvb* locus with those of the *stvb-j* susceptible allele. We then developed DNA markers based on gene structure information and genotyped the resistant and susceptible varieties. The analyses revealed that resistant alleles were highly conserved among the *indica* and *japonica* upland rice varieties, while the specific structures of *stvb-j* were observed only in *japonica* paddy rice varieties. Based on our results, sequence variations around the 5ʹ-end of resistant alleles were classified into five groups. The results showed that the markers effectively predicted resistant and susceptible alleles in rice stripe. The subspecies-dependent RSV resistance in *O. sativa* provides theoretical insights on the application of DNA markers in rice breeding.

## Materials and Methods

### Plant materials

The RSV-resistant variety ‘Rikuto Norin 24’ (‘RN24’) and its progenitor ‘Koshihikari kin-chu-shi SBL1’ (‘KCS1’) carry two RSV resistance genes, *Stva* and *Stvb*. The resistant line ‘Kanto IL 17’ (‘IL17’) is an isogenic line of ‘Koshihikari’ in which RSV resistance was introduced from wild rice (*Oryza officinalis*, IRGC Accession No. 100947; Maeda *et al.* 2007). ‘KCS1’ and ‘IL17’ were developed *via* marker-assisted selection and carried the *Stvb* region of ‘RN24’ and *O. officinalis*, respectively. The RSV-resistant variety ‘St No. 1’ harbors the RSV resistance gene *Stvb-i*, which was derived from the *indica* rice variety ‘Modan’. The RSV-susceptible *japonica* varieties used in this study were ‘Nipponbare’, and ‘Yuukara’. The varieties ‘RN24’, ‘IL17’, ‘St No. 1’, ‘Nipponbare’, and ‘Yuukara’ were used for expression analysis and cloning of genes allelic to *Stvb*. The varieties ‘KCS1’, ‘IL17’, and ‘Yuukara’ were used for genome re-sequencing analysis using next-generation sequencing (NGS) technology. The other *O. sativa* varieties used for genotyping and assessment of RSV resistance are presented in Table 1. A total of 36 rice varieties (Table 1) were used to confirm the response to RSV in the bioassay. Bioassays and assessments of rice stripe resistance were conducted as previously described (Hayano-Saito and Hayashi 2020). The rice core collection of Japanese landraces of the NARO Genebank Project (Ebana *et al.* 2008), world rice core collection of the NARO Genebank Project (Kojima *et al.* 2005), and wild core collection Rank1 (Oryzabase; https://shigen.nig.ac.jp/rice/oryzabase/) were used for genotyping.

### Sequencing and gene annotation

The *Stvb-i* genomic region of St No. 1 (GenBank accession No. LC157868), and Nipponbare (rice annotation project database, RAP-DB, https://rapdb.dna.affrc.go.jp) were used for sequence comparison. Whole genome re-sequencing of ‘KCS1’, ‘IL17’, and ‘Yuukara’ (DDBJ/DRA accession nos. DRX093432, DRX242736 and DRX367773) were performed using an Illumina HiSeq system (Macrogen, Kyoto, Japan, and BGI, Shenzhen, China). Entire sequences of the *Stvb* locus for ‘KCS1’, ‘IL17’, and ‘Yuukara’ were determined by the de novo assembly of the Illumina reads by Platanus v1.2.4 (Kajitani *et al.* 2014) and homology searches by blastn (ncbi-blast-2.6.0+).

Total RNA was extracted from the tissues of six seedlings using TRIZOL Reagent (Invitrogen, California, USA). Seedling-base tissues were used for total RNA extraction to identify the RSV-resistant alleles (*Stvb* and *Stvb-o*), whereas young panicles were used for total RNA extraction to clone the RSV-susceptible alleles (*stvb-j* and *stvb-jy*). The 5ʹ ends of the transcripts were identified using the 5ʹ rapid amplification of cDNA ends (5ʹ-RACE) Full Core Set (Takara Bio, Shiga, Japan). Full-length cDNA of *Stvb*, *Stvb-o* and *stvb-jy* was generated using PrimeScript 1st strand cDNA Synthesis kit (Takara Bio) and KOD Plus (TOYOBO, Osaka, Japan) and sequenced using an ABI3130 genetic analyzer and a BigDye Terminator Cycle Sequencing Kit version 3.1 (Applied Biosystems, Foster City, CA, USA).

The gene structures of *Stvb*, *Stvb-o* and *stvb-jy*, were determined by comparing the cDNA and genome sequences with a *Stvb-i* reference for resistant alleles and *Os11g0541000* (*stvb-j*) for *stvb-jy*, respectively. Domain analysis of the predicted proteins was performed using InterPro (http://www.ebi.ac.uk/interpro/). The nucleotide and amino acid sequences of *Stvb*-allelic genes were aligned using Genetyx-Mac ver. 20 (Genetyx Co., Tokyo, Japan), and Sequencher ver. 5 (Gene Codes Co., Michigan, USA).

### Comparative, ortholog, and homology searches

Genome-wide variation data in TASUKE+ for the NARO Genebank World Rice Core Collection (https://ricegenome-corecollection.dna.affrc.go.jp/) were used for comparative analysis of *Stvb*-allelic genes in rice. Ortholog search among plant species was performed using BLAST in Gramene (http://ensembl.gramene.org/), and homology analysis (similarity and maximum matching score) among the orthologs was performed using Genetyx.

### PCR-based genotyping

Rice varieties were genotyped using four polymorphic markers linked to the *Stvb-i* gene, including two single sequence length polymorphic markers, ST64 and ST71 (Japanese patent application No. 5889626), and two newly developed markers, STrtp (forward: 5ʹ-GATCGGTGGTCTTCTGGACGGC-3ʹ, reverse: 5ʹ-GCGTAGTTCCATACATTGAGACCC-3ʹ) and ST5 (forward: 5ʹ-GGAAGGGTTTTGCAGTTTTGCAG-3ʹ, reverse: 5ʹ-ACCTGGACGAGCTCCATGAGG-3ʹ). The final concentration of each primer in the reaction mixture was 200–400 nM. The PCR cycling using EmeraldAmp (Takara Bio) was as follows: 94°C for 20 s, followed by 94°C for 20 s, 60°C for 20 s, and 72°C for 20 s for 35 cycles. To conduct PCR, template DNA was isolated using the CTAB method (Hayano-Saito and Hayashi 2020), DNA-Suisui S (Rizo Inc., Tsukuba, JAPAN; https://rizo.co.jp), and an alkali treatment method (Wang *et al.* 1993). The PCR products were separated by electrophoresis on a 3.0% (w/v) agarose gel with 1× TAE buffer and visualized by ethidium bromide staining. The PCR product size was measured by sequencing and/or fragment analyses. Sequencing was performed as previously described. Fragment analysis was performed using an ABI3730xl DNA analyzer system (Fasmac Co., Ltd., Atsugi, Japan; https://fasmac.co.jp). Samples for fragment analysis were amplified using the KOD One (Toyobo Co., Ltd. Osaka, Japan).

### Expression analysis

To conduct gene expression analysis, total RNA was extracted from the bases of five rice seedlings. Reverse transcription-polymerase chain reaction (RT-PCR) was performed using a SuperScript III (Invitrogen) and PrimeSTAR HS DNA polymerase (Takara Bio). The *Stvb*-allelic genes were amplified using a sequence-specific forward primer (5ʹ-CTACTCCAAGGACGTCCACTTCC-3ʹ for RSV resistance genes, *Stvb-i*, *Stvb*, and *Stvb-o*; 5ʹ-TTCACCCCGATGGCGTCGC-3ʹ for RSV-susceptible genes, *stvb-j*, and *stvb-jy*) and a common reverse primer (5ʹ-ATGCAAAGCACCATCATCTTACTTG-3ʹ). These primer sets amplify a 5,069-bp fragment from the resistant allele and a 5,354-bp fragment from the susceptible allele. Rice glyceraldehyde 3-phosphate dehydrogenase (*OsGAPDH*; GenBank accession no. AK064960) was used as the reference gene. The PCR products were separated by electrophoresis on a 1.0% (w/v) agarose gel with 1× TAE buffer and visualized by ethidium bromide staining. Quantitative RT-PCR was performed using Roche Lightcycler 96 Real-Time PCR system (Nippon Genetics, Tokyo, Japan) and Thunderbird SYBR qPCR Mix (Toyobo Co., Ltd. Osaka, Japan). The *Stvb*-allelic genes were amplified using a common forward primer (5ʹ-GGCTGGTCTGCTCCAATTAGTC-3ʹ) and a common reverse primer (5ʹ-GCCTCTCTTTGGTGGAAATATCA-3ʹ) located in exon 3. *OsGAPDH* was used as the reference gene. Its primer information was described previously (Hayano-Saito and Hayashi 2020). The mean relative expression levels and standard error of the mean (SEM) were calculated (n = 3) using the statistical package BellCurve for Excel (Social Survey Research Information, Tokyo, Japan).

## Results

### Gene structure analysis of *Stvb*-allelic genes

**Fig. 1.**
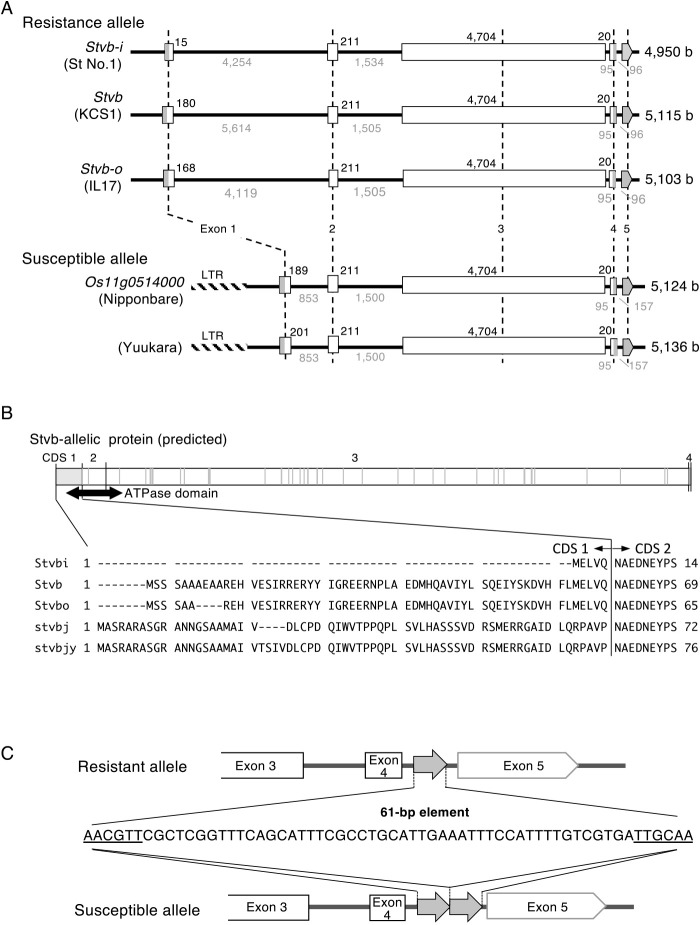
Structure of *Stvb*-allelic genes. A. Squares and pentagons indicate the exons including untranslated region (UTR). Numbers in black and gray represent the length of predicted CDSs and introns, respectively. Gray portions represent predicted UTR regions. Broad hatched portion represents LTR-retrotransposon. B. Outline of predicted *Stvb*-allelic protein and CDS 1 sequence. Gray vertical line represents the position of unmatched amino acid residue among resistant and susceptible alleles. The amino acid sequence of the predicted CDS 1 is represented in single-letter code. The detailed information regarding homology and ATPase domain are shown in Supplemental Tables 1 and 2. C. Sequence of the 61-bp element and its position in RSV-resistant and susceptible alleles. Inverted repeats of the 61-bp element are underlined.

Based on the *Stvb-i* nucleotide sequence, its allelic genes were identified in two RSV-resistant accessions, ‘KCS1’ (*Stvb*; accession no. LC520252) and ‘IL17’ (*Stvb-o*; LC520087), compared with the RSV-susceptible variety ‘Nipponbare’ (*Os11g0514000*; *stvb-j*). All four allelic genes consist of five exons (Fig. 1A). The predicted protein sequences of the genes exhibited high similarity (maximum matching score >97.5%), particularly in exons 2 and 3 (Supplemental Table 1). The sequence of exon 2 (211-bp long), corresponding to the main portion of the ATP-binding domain, which is classified in the histidine kinase/HSP90-like ATPase superfamily (IPR036890), was highly conserved among all four allelic genes (maximum matching score >98.6%; Fig. 1B, Supplemental Table 1). The sequence of exon 1 was predicted to vary among the four alleles, even within the resistant allelic genes (maximum matching score 3.2–93.3%; Fig. 1B, Supplemental Table 1). In particular, in the *stvb-j* susceptible allele, the Gypsy-type LTR-retrotransposon (LOC_*Os11g31490*) insertion led to a complete change in exon 1 from that of the three resistant allelic genes (Fig. 1A, 1B). Another allelic gene of the susceptible variety ‘Yuukara’, *stvb-jy*, exhibited similar gene sequence as *stvb-j*. That is, it contains an LTR-retrotransposon in the upstream region of exon 1, however, it also has a 12-bp insertion and non-sense base substitution in exon 1 and exon 3, respectively (Fig. 1A, 1B).

Another structural difference was the number of unique sequence elements (referred to as a 61-bp element) in the fourth intron. The resistant alleles *Stvb-i*, *Stvb*, and *Stvb-o* carried a single copy, whereas the susceptible alleles, *stvb-j* and *stvb-jy*, carried two tandem copies of this element (Fig. 1C). The result indicated that major structural differences exist between resistant and susceptible alleles at both the 5ʹ- and 3ʹ-ends of the *Stvb* allele.

### *Stvb*-allelic genes in other plant species (orthologs of *Stvb-i*)

To deduce the ancestral structure, *Stvb*-allelic gene sequences were acquired from other plant species as well, including the wild rice variety, by homology search using BLAST in the Gramene database (https://ensembl.gramene.org/). *Stvb-i* orthologs were present in a wide variety of other plant species including monocotyledons and dicotyledons (Supplemental Table 2). The length of exon 2 (211-bp) was highly conserved among the plants and the predicted proteins contained an ATP-binding domain (maximum matching score >71.4%; Supplemental Table 1). The predicted protein sequences of exon 1 (maximum matching score 3.0–96.2%) showed a wider range of similarities than those of exon 3 (maximum matching score 85.9–99.8%) in *Oryza* species (Supplemental Table 1). These results implied that the sequence variation in, or around, exon 1 might serve as a candidate sequence to discriminate between *Stvb*-allelic genes.

### Expression analysis of *Stvb*-allelic genes

Given that young plants are used for biological assessment of stripe resistance (Washio *et al.* 1968c), we examined the expression of *Stvb*-alleles in seedling base tissues. RT-PCR results showed that the resistant *Stvb*-alleles, *Stvb-i*, *Stvb* and *Stvb-o*, were expressed three-times higher than the susceptible alleles (Supplemental Fig. 1). These results suggest that the sequence variation of the promoter region is important for their expression and represents a target candidate for allele-specific markers.

### DNA marker development targeted on *Stvb*-allelic genes

**Fig. 2.**
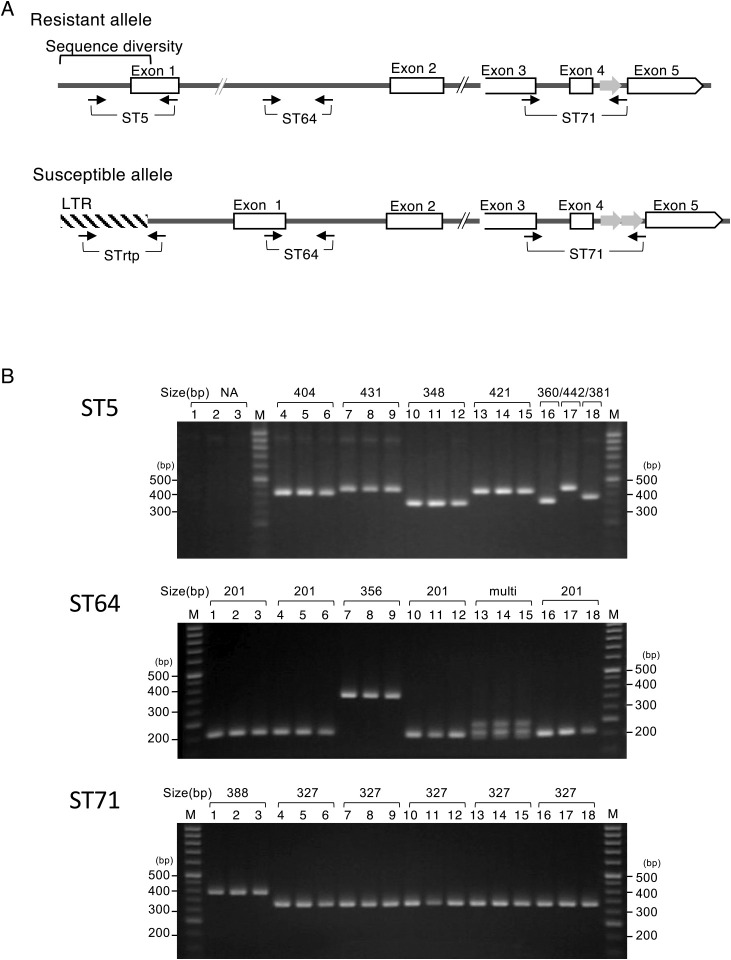
*Stvb*-allele specific markers. A. Positions of ST5, STrtp, ST64 and ST71 markers. Black arrows represent primers with direction. Wide gray arrows represent 61-bp element. B. Amplified fragments of ST5, ST64 and ST71 markers on agarose gel. Variety (lane); Nipponbare (1), Yuukara (2), Koshihikari (3), Kuroka (4), Sensho (5), RN24 (6), St No. 1 (7), Chubu129 (8), Koinoyokan (9), Kasalath (10), Surjumnkhi (11), IL17 (12), Mudgo (13), IR8 (14), IR24 (15), Nato (16), Pe-bi-fun (17), Chukanbohon-no2 (18). Fragment size determined by fragment analysis is presented above the lane number. multi; three fragments of 192 bp, 208 bp and approximately 240 bp. This largest fragment was not detected by fragment analysis. NA, not amplified. M, 50-bp One Step Ladder (Nippon Gene).

Based on the above results, promoter and exon 1 sequences were selected as target sites for DNA markers to discriminate between alleles. To detect sequence variation within the 5ʹ-end region containing the promoter and exon 1, we developed an ST5 marker for the resistant allele and an STrtp marker to detect the LTR-retrotransposon insertion upstream of *stvb-j* (Fig. 2A). Additionally, we developed the ST71 marker (Japanese patent application No. 5889626) to detect the copy number of the 61-bp element (single copy: a 327-bp fragment in the resistant allele, double copies: a 388-bp fragment in the susceptible allele, Figs. 1C, 2) and the ST64 marker (Japanese patent application No. 5889626), which is located within the St No. 1-high polymorphic region in intron 1, to specifically detect the *Stvb-i-*type sequence (Fig. 2A).

Four DNA markers were used to genotype 36 varieties whose response to RSV were identified (Table 1). The markers detected the size of the PCR fragments on an agarose gel and/or DNA fragment analysis (Table 1, Fig. 2B). Genotyping with STrtp, ST5 and ST71 revealed that susceptible varieties (susceptible type, classified as Japonica I) could be discriminated from all resistant varieties. More specifically, STrtp amplified the 343-bp fragment in Japonica I class varieties but not in resistant varieties. Additionally, ST5 showed amplification in all varieties, except in Japonica I class. ST71 can discriminate Japonica I class (a 388-bp fragment) from the other class (a 327-bp fragment) by PCR fragment length. The resistant alleles exhibited varied PCR fragment length by using ST5 and ST64 markers. Accordingly, the genotyping patterns of varieties with strong resistance to RSV were classified into four categories, *Stvb* type (Japonica II), *Stvb-i* type (Indica I), *Stvb-o* type (Indica II), and unknown (Indica III). The PCR fragment of ST5 was amplified as a single fragment in all four resistant categories; similarly, that of ST64 was amplified as a single 201-bp fragment in Japonica II, Indica I, and Indica II, but not in Indica III (Fig. 2B). Although three ST64 fragments were isolated in Indica III via electrophoresis (Fig. 2B), only two smaller fragments were detected by fragment analysis (Table 1); the cause of this discrepancy is unclear. The varieties that showed moderate resistance to RSV showed different PCR fragment sizes in each variety, according to the ST5 marker (Others I).

### Genotyping of *Oryza* species, Japanese and world rice landraces

We genotyped 29 accessions of *Oryza* spp. using three markers. Single or double 61-bp element(s) were detected by ST71 in the *Oryza* species. However, the susceptible STrtp and ST5 genotypes were not shown (Table 2). The results suggested that the accessions in *Oryza* spp. were closely related to the resistance allele structure.

We also genotyped 97 rice accessions, including 41 accessions from the rice core collection of Japanese landraces (JRC) and 56 accessions from the NARO World Rice Core Collection (WRC). The genotyping results using four markers showed no contradiction in all accessions, indicating that the 35 accessions with LTR-retrotransposons in the 5ʹ-end region retained double copies of the 61-bp element (Table 3, Supplemental Table 3). Among the 94 accessions with the subspecies stated, 35 accessions in the susceptible genotype primarily belonged to *japonica*, although other subspecies were present: 21 paddy *japonica*, 4 paddy tropical *japonica*, 4 upland *japonica*, 2 upland tropical *japonica*, 2 paddy *indica*, and 2 upland *indica*. The other 59 accessions, including 44 belonging to *indica*, 10 to upland tropical *japonica*, 3 to paddy tropical *japonica*, and 2 to upland *japonica*, were likely the resistant genotypes (there were no resistant types of paddy *japonica*). Genotyping with ST5 detected other types of sequence patterns in the resistant genotypes, except for Japonica II, Indica I, Indica II, and Indica III. The genotyping results of the four markers were consistent with the RSV-bioassay results, indicating that many *indica* and *japonica* upland varieties exhibited resistance and *japonica* paddy varieties exhibited susceptibility.

The sequences were obtained from TASUKE+ for the NARO Genebank WRC (https://ricegenome-corecollection.dna.affrc.go.jp/) and the translation sequences of exons 2 and 3, conserved regions among *Stvb-i*, *Stvb*, *Stvb-o*, and *stvb-j* were compared. The sequences showed that the two allelic genes created translation stop codons (Table 3, Supplemental Table 3). The two allelic genes, identified in TASUKE+, were classified primarily into two categories genotyped by ST5, one of which was *STV11^IR24^*, and the other was “unknown” (Table 3). These results suggest that ST5 could discriminate two allelic genes with a stop codon in exon 3.

## Discussion

The selection of sites for DNA markers is a crucial aspect of genotyping accuracy. The evolutionary process of genes offers useful information regarding the target sequence. In the present study, we used five *Stvb*-allelic genes, namely, *Stvb-i*, *Stvb*, *Stvb-o*, *stvb-j*, and *stvb-jy*, as RSV-resistant and susceptible sequence templates to classify the gene structure, and subsequently elucidated the unique structure in *japonica* subspecies.

Comparative analysis between resistant and susceptible types of the *Stvb*-allele structure revealed major structural differences between resistant and susceptible alleles in the 5ʹ- and 3ʹ-end sequences. In particular, the LTR-retrotransposon insertion and duplicated 61-bp element were genetic features in *japonica* rice varieties.

Orthologs of *Stvb*-*i* are present in a wide range of plant species (Supplemental Table 2), indicating that they have an important function, especially in monocotyledons. However, rice is not the only host plant for RSV and its insect vector. In fact, 36 other species, including wheat (*Triticum* spp.), maize (*Zea mays*), and grass weeds, have been identified as RSV hosts (Hibino 1989, Ling 1972). The infected plants of non-rice species survive in the same manner as RSV-resistant rice plants; however, the symptoms in non-rice species are milder than in rice (Kisimoto and Yamada 1998, Ling 1972, Shinkai 1962). The results of the current study suggest that orthologs of *Stvb-i* have a resistant function. Thus, the functional allelic genes likely originate in monocotyledons, including *Oryza* species.

In the accessions for *Oryza* species analyzed in this study, no species retained the LTR-retrotransposon in the upstream region of exon 1 (Table 2). Additionally, the *Oryza* AA genome species, containing several *O. rufipogon* accessions, which are considered the ancestors of cultivated rice in Asia, retained a single 61-bp element. In fact, 87.5% of the WRC accessions contain a single 61-bp element and no LTR-retrotransposon (Table 3). Therefore, a resistant-allele type sequence, rather than a *stvb-j* sequence, might represent a main structure in *Oryza* species that is prevalent in cultivated varieties.

Our study indicated that the LTR-retrotransposon insertion in *japonica* varieties of Nipponbare and Yuukara resulted in the loss of RSV resistance via altering the expression, however, the specific biological function of the 61-bp element has not been defined. Nevertheless, genotyping analyses with STrtp and ST71 distinguished susceptible allele (Japonica I) from resistant alleles (Table 1) and revealed that all *japonica* paddy varieties contain a *stvb-j* type sequence (Table 3). This result strongly suggests that major RSV susceptible alleles in *japonica* paddy varieties have occurred, including LTR-retrotransposon insertion and 61-bp element duplication, during the evolutionary process.

The ST5 marker developed from 5ʹ-end sequences (exon 1 and the upper sequence of exon 1) can classify resistant alleles into five categories (Table 1). The variation of 5ʹ-end sequences was used to analyze resistant allele evolution as exons 2 and 3 among *Stvb* allelic genes exhibited high similarity (Supplemental Table 1). Among the 13 JRC and 49 WRC accessions that were categorized as resistant type genotyped by ST5, 13 JRC and 36 WRC accessions were grouped into Japonica II, Indica II, and Indica III (Table 3). This finding suggests that the *Stvb*-alleles might have relatively low diversity. However, we were unable to clarify the relationship between the genetic background of three varieties that conferred moderate resistance and their phenotype. Given that the three WRC accessions had the same length of ST5 PCR fragment as the three moderate resistant varieties, minor variations likely occurred (Table 3). Hence, the sequence region surrounding ST5 is a useful target for assessing the genetic background of the resistant type varieties.

The results of the genetic analyses suggest that a unique evolution process occurred in *japonica* paddy rice varieties. Diverse rice varieties were grown until breeding systems were established (Bos 1992, Kushibuchi 1997), resulting in the maintenance of RSV-resistant and RSV-susceptible lines for a long time. In Japan, this supposition was supported by the JRC genotyping results, in that 11 *japonica*/*tropical japonica* upland varieties were *Stvb* type, and 19 *japonica* paddy varieties were *stvb-j* type (Table 3). However, rice cultivation has changed over the past 100 years. *Japonica* paddy rice, which is vulnerable to RSV, was selected as the leading variety in Japan. Numerous newly bred *japonica* paddy varieties have superior agronomic traits but are susceptible to RSV (Washio *et al.* 1967, 1968c). Owing to the expansion of these new varieties, genetic diversity at the *Stvb* locus may have been rapidly lost in paddy fields in Japan. This may account for the RSV outbreaks reported in the mid-twentieth century in Japan, although several other factors also contributed to the phenomena (Hibino 1996, Jones 2008). Rice cultivation has probably followed a similar history in Korea (Cho *et al.* 2013, Hibino 1996). In eastern China, an outbreak occurred in the early 1960s and rice stripe re-emerged as a serious threat after 2000 (He *et al.* 2016, Wang *et al.* 2008, Yu *et al.* 2014), coinciding with the replacement of *indica* hybrid rice varieties with *japonica* (Otuka 2013). The rice stripe epidemics in these countries are assumed to result from the targeted selection of *stvb-j* type of *japonica* rice in breeding programs.

The selection and cultivation of the current leading variety of *stvb-j* type of *japonica* paddy rice involve high risks as it perpetuates RSV susceptibility. Therefore, it is essential to develop RSV-resistant varieties to compensate the allele. One useful strategy is the application of DNA markers in rice breeding programs (Kang *et al.* 2019). Our developed marker is a useful tool to identify *stvb-j* type varieties and candidate resistant varieties.

Our results suggest that numerous genetic resources have the potential to act as donors of RSV resistance (Table 3). Sequence data, including the genome-wide variation data, are available on open-source repositories, making them powerful tools for deducing gene function. However, data are limited for genes with structures that are the same as those of the GWAS reference variety, for example, ‘Nipponbare’ in the case of rice. Using the data in the case of resistance genes, such as *Stvb*-allelic genes, can be problematic due to the lack of sequence information in the reference genome. Hence, it is often challenging to deduce genetic alterations. However, amino acid changes could contribute to the loss of the resistance function at *Stvb*-allelic genes. The protein sequence data could be used to predict whether the *Stvb* alleles have a function in combination with the genotyping results of the marker.

Overall, this study provides novel insights on rice breeding to control RSV. DNA markers developed from the results of the genetic background analyses can increase the accuracy in differentiating between alleles that confer resistance or susceptibility to rice stripe at the *Stvb* locus. Based on our findings, the disease in current leading *japonica* varieties can be controlled by marker-assisted selection within a traditional breeding program (Hayano-Saito and Hayashi 2020, Maeda and Hayano-Saito 2016). The development and cultivation of *japonica* rice varieties with stripe-resistant genes will ensure food security.

## Author Contribution Statement

YH designed the experiments. YH and KH performed the experiments, analyzed the data, and wrote the manuscript. YK analyzed the NGS data and information. HM produced RSV-resistant varieties Koshihikari kin-chu-shi SBL1 and Kanto IL 17. YH and HM assessed the stripe resistance using a bioassay.

## Supplementary Material

Supplemental Figure

Supplemental Tables

## Figures and Tables

**Table 1.** Classification of rice varieties by using DNA markers linked to the *Stvb*-allelic genes

Varieties	Response to RSV	STrtp (bp)*^a^*	ST5 (bp)*^b^*	ST64 (bp)*^b^*	ST71 (bp)*^b^*	No. of 61-bp element	Gene	Type of *Stvb*-allele
Nipponbare, Yuukara, Koshihikari, Hokkaiakage*^c^*, Bouzumochi*^c^*, Katakutara	S	343	nd	201	388	2	*stvb-j* *stvb-jy*	Japonica I
Kuroka, RN11, RN22*^c^*, RN24, Zenith*^c^*, Sensho*^c^*, Shirohige*^c^*, KCS1 (RN24)	R	nd	404	201	327	1	*Stvb*	Japonica II
Modan, St No.1(Modan), Tachiaoba (Modan)*^d^*, Chubu129 (Modan)*^d^*, Koinoyokan (Modan)*^d^*	R	nd	431	356	327	1	*Stvb-i*	Indica I
Kasalath, Karalath, Surjumnkhi, IL17 (*O.officinalis*), Minamiyutaka (RINX89)*^d^*, Calorina	R	nd	348	201	327	1	*Stvb-o*	Indica II
Mudgo, IR8, IR24, Hoshiaoba (IR24), Hokuriku193 (IR24)*^d^*, Habataki (IR24)*^d^*, Oonari (IR24)*^d^*	R	nd	421	208/192*^e^*	327	1	Unknown	Indica III
Nato, Saturn Pe-bi-fun Chukannbohon-no2	M	nd	360 442 381	201	327	1	Unknown	Others I

*^a^* Data of Sequencing analysis data. *^b^* Data of fragment analysis. *^c^* Referred from Washio *et al.* (1968c). *^d^* Referred from the rice variety database (https://ineweb.narcc.affrc.go.jp/). *^e^* Two fragments were detected by fragment analysis (*see* gel-electrophoresis image shown in Fig. 2B). Response to RSV: R, resistant; M, moderate; S, susceptible. The resistance donor variety is shown in parentheses after the variety name.

**Table 2.** Genotypes of wild rice species determined using DNA markers linked to the *Stvb*-allelic genes

No.*^a^*	Species	Genome	Origin (country)	STrtp	ST5	ST71
W0652	*O. barthii*	AA	Sierra Leone	–	+	1
W1588	*O. barthii*	AA	Cameroun	–	+	(1)
W1169	*O. glumaepatula*	AA	Cuba	–	+	1
W2145	*O. glumaepatula*	AA	Brazil	–	+	1
W1625	*O. meridionalis*	AA	Australia	–	+	1
W1635	*O. meridionalis*	AA	Australia	–	+	1
W0106	*O. rufipogon*	AA	India	–	+	1
W0120	*O. rufipogon*	AA	India	–	+	1
W1866	*O. rufipogon*	AA	Thailand	–	+	1
W1921	*O. rufipogon*	AA	Thailand	–	+	1
W2003	*O. rufipogon*	AA	India	–	+	1
W1514	*O. punctata* (2X)	BB	Kenya	–	+	3
W1024	*O. punctata* (4X)	BBCC	Ghana	–	+	1
W1331	*O. minuta*	BBCC	Philippines	–	+	1
W0002	*O. officinalis*	CC	Thailand	–	+	–
W1830	*O. officinalis*	CC		–	+	(1)
W1805	*O. rhizomatis*	CC	Sri Lanka	–	+	(1)
W0017	*O. alta*	CCDD	Surinam	–	–	(1)
W1182	*O. alta* or *O. latifolia*	CCDD	British Guiana	–	+	1
W0613	*O. grandiglumis*	CCDD	Brazil	–	+	(1)
W1194	*O. grandiglumis*	CCDD	Brazil	–	+	3
W2220	*O. grandiglumis*	CCDD	Brazil	–	+	1
W2200	*O. latifolia*	CCDD	Brazil	–	+	1
W1166	*O. latifolia*	CCDD	Mexico	–	+	3
W1197	*O. latifolia*	CCDD	Colombia	–	+	3
W0008	*O. australiensis*	EE	Australia (?)	–	+	3
W1401	*O. brachyantha*	FF	Sierra Leone	–	–	(1)
W1711	*O. brachyantha*	FF	Cameroun	–	+	3
W0001	*O. ridleyi*	HHJJ	Thailand	–	+	–

The genotypes of 14 representative wild rice species (AA, BB, CC, BBCC, CCDD, EE, FF, and HHJJ genomes) are indicated. +, fragment(s) amplified by ST5 marker were shown to have various sizes; 1, a 327-bp band of ST71 (*see* Fig. 2); (1) a faint and indistinct 327-bp band of ST71; 3, both 327- and 388-bp bands of ST71; –, no fragment was detected. *^a^* The accession numbers of the species were obtained from Oryzabase (https://shigen.nig.ac.jp/rice/oryzabase/).

**Table 3.** Genotypes of Japanese and world landraces by using DNA markers linked to the *Stvb*-allelic genes

ID*^a^*					Paddy/Upland	Subspecies	STrtp*^b^*	ST5*^c^* size (bp)	ST71*^d^*	Gene*^e^*
JRC 40	JRC 43				upland	*Indica*	A	—	2	*stvb-j*
JRC 42	WRC 10				paddy					
JRC 12	JRC 13				upland	*Tropical Japonica*				
WRC 47	WRC 48	WRC 52	WRC 53		paddy					
JRC 18	JRC 29	JRC 49	JRC 53		upland	*Japonica*				
JRC 17	JRC 19	JRC 20	JRC 21	JRC 23	paddy					
JRC 24	JRC 26	JRC 27	JRC 31	JRC 32						
JRC 33	JRC 34	JRC 35	JRC 36	JRC 38						
JRC 39	JRC 45	JRC 51	JRC 54	WRC 67						
WRC 68										
JRC 47	JRC 48				upland	*Japonica*	—	404	1	*Stvb*
WRC 45	WRC 49				paddy	*Tropical Japonica*				
JRC 01	JRC 03	JRC 04	JRC 05	JRC 07	upland					
JRC 08	JRC 10	JRC 11	JRC 14	WRC 51						
WRC 24					paddy	—	—	348	1	*Stvb-o* *STV11^KAS^*
JRC 41	JRC 44	WRC 02	WRC 04	WRC 20	paddy	*Indica*				
WRC 26	WRC 31	WRC 32	WRC 34	WRC 37						
WRC 38	WRC 44	WRC 58	WRC 66	WRC 97						
WRC 23**					paddy	—	—	421	1	*STV11^IR24^*
WRC 03**	WRC 05**	WRC 06**	WRC 07	WRC 09**	paddy	*Indica*				
WRC 11	WRC 12**	WRC 13	WRC 15**	WRC 16**						
WRC 21**	WRC 35	WRC 60**	WRC 61	WRC 62**						
WRC 63	WRC 64	WRC 100								
WRC59**					paddy	*Indica*	—	433/421	1	Unknown
WRC 27*	WRC 28*	WRC 29*	WRC 30*	WRC 39*	paddy	*Indica*	—	449	1	
WRC 65*										
WRC 18					paddy	*Indica*	—	442	1	
WRC 17					paddy	*Indica*	—	381	1	
WRC 22					paddy	—	—	360	1	
WRC 46					paddy	*Tropical Japonica*				
WRC 36	WRC 41				paddy	*Indica*	—	334	1	

A total of 97 (Japanese 41 and world 56) rice landraces were genotyped using the *Stvb-i*–linked markers. Prefix JRC and WRC represent Japanese collection and world collection, respectively. *,**, allelic genes were predicted to have stop codons at the different positions in exon 3 in TASUKE+. *^a^* The ID was obtained from the NARO Genebank Project (Tsukuba, Japan). *^b^* STrtp marker gives approximately 343-bp single (A) or no (–) fragment. *^c^* The size of the ST5-amplified fragment was measured by fragment analysis. *^d^* The gnotypes of ST71, 1 and 2, represent single copy and two copies of the 61-bp element, respectively. *^e^* A corresponding gene to the group; *STV11^KAS^* and *STV11^IR24^* were referred to Wang *et al.* (2011) and Zhang *et al.* (2011). The detailed data was shown in Supplemental Table 3.
