# Supplementary material for: Comparative analyses of Stvb-allelic genes reveal japonica specificity of rice stripe resistance in Oryza sativa
Source: Breed Sci. 2022 Dec 6;72(5):333–42. doi: 10.1270/jsbbs.22027 (PMC9895804; doi:10.1270/jsbbs.22027)
Supplement: Supplementary file 1 — Supplemental Figure [file 72_333_s1.pdf]

doi: 10.1270/jsbbs.22027

A

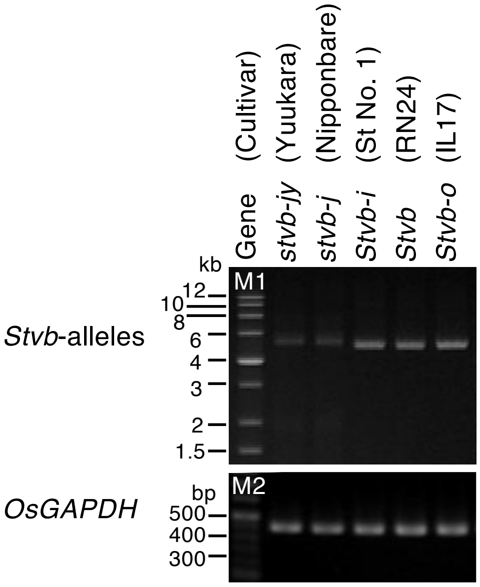

B

Variety	Yuukara	Nipponbare	St No.1	RN24	IL17
Gene	*stvb-jy*	*stvb-j*	*Stvb-i*	*Stvb*	*Stvb-o*
Average (× 10^{-4})	1.18	1.13	3.84	4.78	3.78
SEM (× 10^{-4})	0.19	0.34	0.36	1.25	0.39

Supplemental Fig. 1. Expression of *Stvb*-allelic genes in the 2.5-leaf staged seedlings. *A*. Amplified fragment on 1% agarose gel (5,354-bp long from susceptible allele; 5,069-bp long from resistant allele). M1, 0.5–12-kb perfect DNA marker (Novegen, Darmstadt, Germany); M2, 50-bp OneSTEP Ladder (Nippon Gene Co. Ltd.). *OsGAPDH* was used as a reference (452-bp long) . *B*. Relative expression levels of the *Stvb*-alleles to reference *OsGAPDH* gene with the SEM (n=3).
