# Supplementary material for: Comparative analyses of Stvb-allelic genes reveal japonica specificity of rice stripe resistance in Oryza sativa
Source: Breed Sci. 2022 Dec 6;72(5):333–42. doi: 10.1270/jsbbs.22027 (PMC9895804; doi:10.1270/jsbbs.22027)
Supplement: Supplementary file 2 — Supplemental Tables [file 72_333_s2.pdf]

Hayashi et al./Breeding Science
doi: 10.1270/jsbbs.22027

Supplemental Table 1. Homology of *Stvb*–allelic proteins and the orthologs

			Upper stream region of 211-bp exon																
		Transcript No.	1	2	3	4	5	6	7	8	9	10	11	12	13	14	15	16	17
No.	Transcript	Species size(aa)	5	60	56	63	78	78	113	67	59	54	63	61	57	54	59	58	245
1	Stvbi	*Oryza sativa indica* Group	*	100	100	80.0	80.0	40.0	80.0	80.0	100	100	100	100	100	100	100	100	100
2	Stvb	*Oryza sativa japonica* Group	8.3	*	98.2	68.0	58.3	64.4	59.2	68.0	96.6	98.1	95.0	93.3	96.4	96.2	96.6	96.5	88.1
3	Stvbo	*Oryza officinalis*	8.9	93.3	*	60.5	61.3	59.0	57.1	59.0	96.4	98.1	96.4	94.6	94.6	96.2	96.4	96.4	89.0
4	stvbj (Os11t0514000-01)	*Oryza sativa japonica* Group	3.2	17.2	18.2	*	76.0	76.0	76.0	100	55.5	66.6	68.0	65.1	64.1	69.4	66.6	46.6	57.1
5	OBART11G14820.1	*Oryza barthii*	3.9	16.3	15.0	37.4	*	98.7	92.3	82.0	66.6	59.5	56.8	55.1	57.7	57.1	57.4	54.3	51.2
6	KN541612.1_FGT003	*Oryza longistaminata*	3.9	15.2	13.9	38.5	96.2	*	89.7	82.0	64.4	57.1	54.9	53.0	55.5	54.1	59.5	52.1	52.2
7	OMERI11G12160.1	*Oryza meridionalis*	3.6	13.4	14.3	28.0	58.0	59.8	*	82.0	55.8	63.1	59.5	55.5	60.9	60.5	60.4	65.2	58.0
8	ONIVA11G14270.1	*Oryza nivara*	3.0	22.1	20.6	94.0	40.0	41.1	30.2	*	55.5	56.8	68.0	65.1	56.8	69.4	66.6	54.5	58.2
9	OPUNC11G12410.1	*Oryza punctata*	6.8	81.7	81.4	14.1	13.9	15.2	12.5	16.2	*	100	96.6	98.3	98.2	98.1	100	96.5	91.3
10	HORVU5Hr1G085220.35	*Hordeum vulgare*	9.3	76.7	80.4	20.9	12.7	13.9	12.5	19.4	72.9	*	100	100	100	98.1	100	100	96.2
11	OQU83490	*Sorghum bicolor*	7.9	79.4	79.4	15.6	17.5	18.8	16.1	14.7	84.1	76.2	*	93.4	96.4	98.1	96.6	96.5	87.0
12	TraesCS5A02G339700.1	*Triticum aestivum*	8.2	82.0	77.1	15.9	12.7	13.9	14.3	21.7	75.4	80.3	84.1	*	100	98.1	98.3	96.5	91.6
13	TRIDC2BG072840.4	*Triticum dicoccoides*	8.8	78.3	79.0	14.3	11.4	12.7	12.5	20.3	74.6	86.0	81.0	93.4	*	98.1	98.2	98.2	96.4
14	TRITD5Bv1G178410.1	*Triticum turgidum*	9.3	76.7	82.1	21.5	12.7	13.9	12.5	20.9	76.3	96.3	79.4	82.0	86.0	*	98.1	98.1	94.3
15	Zm00001eb114050_T002	*Zea mays*	8.5	83.3	83.1	17.2	15.2	16.5	14.3	21.1	78.0	76.3	82.5	80.3	79.7	79.7	*	98.2	93.1
16	Ma09_t01130.1	*Musa acuminata*	6.9	73.3	74.1	15.4	13.9	15.2	12.5	16.4	71.2	74.1	71.4	70.5	72.4	77.6	73.3	*	92.9
17	AT3G48770.2	*Arabidopsis thaliana*	1.6	15.0	16.6	10.2	12.2	11.8	14.3	11.0	16.3	14.2	15.9	15.0	13.8	13.8	15.9	15.9	*

			211-bp exon																
		Transcript No.	1	2	3	4	5	6	7	8	9	10	11	12	13	14	15	16	17
No.	Transcript	Species size(aa)	71	71	71	71	71	71	71	71	71	71	71	71	71	71	71	71	71
1	Stvbi	*Oryza sativa indica* Group	*	100	100	100	100	100	100	100	100	98.5	98.5	98.5	98.5	97.1	98.5	98.2	94.2
2	Stvb	*Oryza sativa japonica* Group	98.6	*	100	100	100	100	100	100	100	95.7	98.5	98.5	98.5	97.1	98.5	92.8	94.2
3	Stvbo	*Oryza officinalis*	98.6	100	*	100	100	100	100	100	100	95.7	98.5	98.5	98.5	97.1	98.5	92.8	94.2
4	stvbj (Os11t0514000-01)	*Oryza sativa japonica* Group	98.6	100	100	*	100	100	100	100	100	95.7	98.5	98.5	98.5	97.1	98.5	92.8	94.2
5	OBART11G14820.1	*Oryza barthii*	98.6	95.8	97.1	97.1	*	100	100	100	100	95.7	98.5	98.5	98.5	97.1	98.5	92.8	94.2
6	KN541612.1_FGT003	*Oryza longistaminata*	98.6	95.8	97.1	97.1	97.1	*	100	100	100	95.7	98.5	98.5	98.5	97.1	98.5	92.8	94.2
7	OMERI11G12160.1	*Oryza meridionalis*	100	97.2	98.6	98.6	98.6	98.6	*	100	100	95.7	98.5	98.5	98.5	97.1	98.5	92.8	94.2
8	ONIVA11G14270.1	*Oryza nivara*	98.6	98.5	100	100	97.1	97.1	98.6	*	100	95.7	98.5	98.5	98.5	97.1	98.5	92.8	94.2
9	OPUNC11G12410.1	*Oryza punctata*	100	97.2	98.6	98.6	98.6	98.6	100	98.6	*	95.7	98.5	98.5	98.5	97.1	98.5	92.8	94.2
10	HORVU5Hr1G085220.35	*Hordeum vulgare*	88.6	85.9	87.1	87.1	87.1	87.1	88.5	87.1	88.6	*	97.1	97.1	97.1	95.7	97.1	92.8	97.1
11	OQU83490	*Sorghum bicolor*	84.3	81.7	82.9	82.9	82.9	82.9	84.3	82.9	84.3	81.4	*	100	100	98.5	100	92.8	97.1
12	TraesCS5A02G339700.1	*Triticum aestivum*	92.9	90.1	91.4	91.4	91.4	91.4	92.9	91.4	92.9	90.0	84.3	*	100	98.5	100	94.2	97.1
13	TRIDC2BG072840.4	*Triticum dicoccoides*	92.9	90.1	91.4	91.4	91.4	91.4	92.9	91.4	92.9	90.0	84.3	100	*	98.5	100	94.2	97.1
14	TRITD5Bv1G178410.1	*Triticum turgidum*	91.4	88.7	90.0	90.0	90.0	90.0	91.4	90.0	91.4	88.6	84.3	98.6	98.6	*	98.5	92.8	95.7
15	Zm00001eb114050_T002	*Zea mays*	84.3	81.7	82.9	82.9	82.9	82.9	84.3	82.9	84.3	81.4	91.4	84.3	84.3	84.3	*	92.8	97.1
16	Ma09_t01130.1	*Musa acuminata*	74.3	71.8	72.9	72.9	72.9	74.3	74.3	72.9	74.3	72.9	77.1	72.9	72.9	71.4	77.1	*	91.4
17	AT3G48770.2	*Arabidopsis thaliana*	78.6	76.1	77.1	77.1	77.1	77.1	78.6	77.1	78.6	75.7	77.1	75.7	75.7	74.3	77.1	74.3	*

			Down stream region of 211-bp exon																
		Transcript No.	1	2	3	4	5	6	7	8	9	10	11	12	13	14	15	16	17
No.	Transcript	Species size(aa)	1573	1573	1573	1573	1579	1573	1622	1573	1579	1489	1449	1575	1575	1585	1570	1566	1583
1	Stvbi	*Oryza sativa indica* Group	*	99.5	99.6	99.6	99.2	99.4	98.8	99.4	98.8	94.1	94.3	95.1	95.0	94.5	93.9	88.9	86.5
2	Stvb	*Oryza sativa japonica* Group	97.5	*	99.8	99.8	99.0	99.1	99.0	99.9	97.8	93.9	94.1	95.0	94.9	94.2	93.6	89.4	86.2
3	Stvbo	*Oryza officinalis*	97.5	99.2	*	99.9	99.2	99.3	99.1	99.7	98.9	94.1	94.2	95.1	95.1	94.4	93.8	89.5	86.4
4	stvbj (Os11t0514000-01)	*Oryza sativa japonica* Group	97.5	99.6	99.3	*	99.2	99.3	99.0	99.8	98.8	94.1	94.2	95.1	95.0	94.3	93.8	89.7	86.3
5	OBART11G14820.1	*Oryza barthii*	91.2	90.8	90.8	90.8	*	99.2	98.2	99.0	98.8	93.5	94.2	95.0	95.0	94.2	93.5	89.0	77.0
6	KN541612.1_FGT003	*Oryza longistaminata*	97.8	97.1	97.1	97.1	91.1	*	98.4	99.1	98.7	94.2	94.3	95.0	94.9	94.5	94.0	89.8	86.4
7	OMERI11G12160.1	*Oryza meridionalis*	92.3	93.4	99.4	93.5	85.9	91.9	*	98.9	98.3	94.1	93.9	94.6	94.5	91.4	92.9	89.3	85.6
8	ONIVA11G14270.1	*Oryza nivara*	97.5	99.8	99.2	99.6	90.8	97.1	93.5	*	98.6	93.9	94.0	94.9	94.9	94.2	93.6	89.4	86.1
9	OPUNC11G12410.1	*Oryza punctata*	93.0	92.6	92.6	92.6	87.3	92.8	87.9	92.6	*	94.5	94.4	95.3	95.2	94.8	94.0	89.7	86.1
10	HORVU5Hr1G085220.35	*Hordeum vulgare*	68.4	68.2	68.4	68.3	64.4	68.5	66.3	68.3	68.5	*	94.3	94.9	94.9	98.1	93.5	88.8	85.4
11	OQU83490	*Sorghum bicolor*	67.9	67.3	67.4	67.3	63.7	67.5	65.3	67.2	67.8	68.7	*	95.7	95.6	94.0	97.6	89.1	86.3
12	TraesCS5A02G339700.1	*Triticum aestivum*	74.8	74.3	74.5	74.3	70.2	74.3	70.6	74.3	74.9	71.1	67.1	*	99.8	95.0	94.9	89.0	86.0
13	TRIDC2BG072840.4	*Triticum dicoccoides*	74.5	74.0	74.1	74.0	69.8	74.0	70.4	74.0	74.7	70.9	66.9	99.6	*	95.0	94.7	89.0	86.0
14	TRITD5Bv1G178410.1	*Triticum turgidum*	71.4	71.2	71.4	71.3	67.3	71.4	67.5	71.2	71.3	85.1	64.5	74.0	73.7	*	93.8	88.9	85.2
15	Zm00001eb114050_T002	*Zea mays*	73.4	78.0	73.1	73.1	69.0	73.1	69.4	72.9	73.1	65.6	80.5	71.2	71.1	69.7	*	88.9	86.2
16	Ma09_t01130.1	*Musa acuminata*	55.3	55.0	55.0	54.9	52.5	55.5	52.2	54.9	55.3	50.9	50.0	54.2	54.1	52.7	54.8	*	86.7
17	AT3G48770.2	*Arabidopsis thaliana*	47.5	47.4	47.5	47.3	45.4	47.4	45.5	47.3	46.9	44.5	43.1	47.1	47.1	46.3	46.4	46.8	*

Upper part: Simirality (%)

Lower part: maximum matching (%)

Supplemental Table 2. Orthologs of the *Stvb* -allelic genes

Gene	Trancript ID	Species	Transcript size (AA)	Position of IPR036890 superfamily		CDS length (bp)																								
				start	end	1	2	3	4	5	6	7	8	9	10	11	12	13	14	15	16	17	18	19	20	21	22	23	24	25
Os11g0514000	Os11t0514000-01	*Oryza sativa japonica* Group	1707	66	245	189	**211**	4704	20																					
allele	Stvbi	*Oryza sativa indica* Group	1645	1	170	15	**211**	4704	20																					
allele	Stvb	*Oryza sativa japonica* Group	1704	12	225	180	**211**	4704	20																					
allele	Stvbo	*Oryza officinalis*	1700	8	221	168	**211**	4704	20																					
BGIOSGA033928	BGIOSGA033928-TA	*Oryza sativa Indica* Group	2351	45/782	225/973	189	**211**	1035	345	455	189	**211**	1258	3166																
OBART11G14820	OBART11G14820.1	*Oryza barthii*	1728	-	-	234	**211**	65	85	3494	1078	20																		
OB0111G10020	OB0111G10020.1	*Oryza brachyantha*	1671	44	224	186	**211**	653	483	816	312	2060	292																	
KN541612.1_FG003	KN541612.1_FGT003	*Oryza longistaminata*	1722	103	240	234	**211**	4704	20																					
OMERI11G12160	OMERI11G12160.1	*Oryza meridionalis*	1805	138	260	336	**211**	4435	236	23	51	496																		
ONIVA11G14270	ONIVA11G14270.1	*Oryza nivara*	1711	93	229	201	**211**	4704	20																					
OPUNC11G12410	OPUNC11G12410.1	*Oryza punctata*	1700	34	221	177	**211**	4717																						
ORUFI11G15810	ORUFI11G15810.3	*Oryza rufipogon*	2412	204	352	201	108	**211**	17	**211**	17	219	123	185	172	291	296	301	162	1145	561	861	138	1018	699	101	15	38	68	79
ORGLA11G0126400	ORGLA11G0126400.1	*Oryza glaberrima*	1705	-	-	200	**211**	4697	10																					
PAHAL_8G185600	PVH34281	*Panicum hallii FIL2*	1738	40	227	282	**211**	4791	509																					
PAHAL_8G185600	PVH34281	*Panicam hallii FIL2*	1738	40	227	282	**211**	4791	509																					
GQ55_8G182500	PUZ44966	*Panicum hallii HAL2*	1758	45	231	210	**211**	4838	727																					
BRADI_4g18296v3	PNT63606	*Brachypodium distachyon*	1535	-	-	**211**	2034	1215	250	330	292	94	182																	
EJB05_27763	TVU25271	*Eragrostis curvula*	1701	39	216	192	**211**	4703																						
EJB05_27770	TVU25278	*Eragrostis curvula*	1701	39	216	192	**211**	4703																						
HORVU5Hr1G085220	HORVU5Hr1G085220.35	*Hordeum vulgare*	1614	29	199	162	**211**	4439	219																					
LPERR11G11650	LPERR11G11650.1	*Leersia perrieri*	1784	166	290	180	246	**211**	4720																					
Sspon.06G0014460-2C	Sspon.06G0014460-2C-mRNA-1	*Saccharum spontaneum*	1699	37	216	284	**211**	4697																						
SEVIR_8G138100v2	TKW00831	*Setaria viridis*	1745	45	223	210	**211**	529	4270	18																				
Ma09_g01130	Ma09_t01130.1	*Musa acuminata*	1692	30	203	197	**211**	4773																						
SETIT_027723mg	KQK94707	*Setaria italica*	1683	31	209	168	**211**	1004	3669																					
SETIT_028332mg	KQK94695	*Setaria italica*	1272	41	219	198	**211**	3261	146																					
SORBI_3005G124100	KXG28449	*Sorghum bicolor*	1715	38	210	408	**211**	4719	748																					
SORBI_3005G124200	KXG28450	*Sorghum bicolor*	1714	38	210	265	**211**	4716	679																					
SORBI_3005G124500	OQU83490	*Sorghum bicolor*	1583	38	210	261	**211**	4349																						
SORBI_3005G125000	OQU83492	*Sorghum bicolor*	1705	38	216	426	**211**	5495																						
SORBI_3005G124500	OQU83490	*Sorghum bicolor*	1583	38	210	261	**211**	4349																						
SORBI_3005G125000	OQU83492	*Sorghum bicolor*	1705	38	216	426	**211**	5495																						
TraesCS2A02G479300	TraesCS2A02G479300.1	*Triticum aestivum*	1695	38	200	114	48	**211**	4715																					
TraesCS2B02G503700	TraesCS2B02G503700.1	*Triticum aestivum*	1707	36	208	183	**211**	4710	436																					
TraesCS5A02G339700	TraesCS5A02G339700.1	*Triticum aestivum*	1702	31	190	132	**211**	1916	2289	561																				
TraesCS5B02G338300	TraesCS5B02G338300.1	*Triticum aestivum*	1718	29	199	162	**211**	4784																						
TRIDC2BG072840	TRIDC2BG072840.4	*Triticum dicoccoides*	1703	32	204	171	**211**	4170	19																					
TRIDC5AG049930	TRIDC5AG049930.5	*Triticum dicoccoides*	1685	6	177	95	**211**	4734	55																					
TRIDC2AG067660	TRIDC2AG067660.7	*Triticum dicoccoides*	1475	41	202	170	**211**	4094																						
TRITD5Bv1G178410	TRITD5Bv1G178410.1	*Triticum turgidum*	1710	29	199	162	**211**	4740	20																					
TRIUR3_17742	TRIUR3_17742-T1	*Triticum urartu*	1686	29	200	162	**211**	1838	2847																					
Zm00001eb114050	Zm00001eb114050_T002	*Zea mays*	1700	34	206	304	**211**	4695	56																					
AT3G48770	AT3G48770.2	*Arabidopsis thaliana*	1899	221	392	390	147	198	211	4755	101	97																		
GSCOC_T00010617001	CDP20873	*Coffea canephora*	1687	30	203	165	**211**	4565	32	226																				
Csa_1G000750	KGN63452	*Cucumis sativus*	1703	29	217	192	**211**	4929																						
CCACVL1_08596	OMO88010	*Corchorus capsularis*	1906	29	204	162	**211**	4780	56	512																				
RchiOBHm_Chr7g0189261	PRQ16901	*Rosa chinensis*	1700	31	222	285	**211**	4911																						
CEY00_Acc22096	PSR95961	*Actinidia chinensis*	1702	29	199	256	**211**	4916																						

Supplemental Table 3. Genotypes of Japanese and world rice landraces determined using DNA markers linked to *Stvb* -allelic genes

ID[a]	Cultivar name	Origin[b]	Paddy/Upland	Subspecies	STrtp	ST5[c]	ST64	ST71
JRC 01	Gaisen Mochi	unknown	upland	*Tropical Japonica*	–	404	C	1
JRC 03	Hinode	Kinki region	upland	*Tropical Japonica*	–	404	C	1
JRC 04	Sensho	Tokyo	upland	*Tropical Japonica*	–	404	C	1
JRC 05	Yamada bake	Kagoshima	upland	*Tropical Japonica*	–	404	C	1
JRC 07	Iruma nishiki	Saitatama	upland	*Tropical Japonica*	–	404	C	1
JRC 08	Okka moroshi	unknown	upland	*Tropical Japonica*	–	404	C	1
JRC 10	Hirayama	Tokyo	upland	*Tropical Japonica*	–	404	C	1
JRC 11	Kahei	Kagoshima	upland	*Tropical Japonica*	–	404	C	1
JRC 12	Oiran	Kumamoto	upland	*Tropical Japonica*	A	–	C	2
JRC 13	Bouzu mochi	Oita	upland	*Tropical Japonica*	A	–	C	2
JRC 14	Megura mochi	Kanto-Tosan region	upland	*Tropical Japonica*	–	404	D	1
JRC 17	Akage	Yamagata	paddy	*Japonica*	A	–	D	2
JRC 18	Hassokuho	unknown	upland	*Japonica*	A	–	D	2
JRC 19	Wataribune	Shiga	paddy	*Japonica*	A	–	D	2
JRC 20	Hosogara	Aomori	paddy	*Japonica*	A	–	D	2
JRC 21	Akamai	Kochi	paddy	*Japonica*	A	–	C	2
JRC 23	Ishijiro	Toyama	paddy	*Japonica*	A	–	D	2
JRC 24	Joushuu	Yamagata	paddy	*Japonica*	A	–	C	2
JRC 26	Aikoku	Fukui	paddy	*Japonica*	A	–	D	2
JRC 27	Ginbouzu	Ishikawa	paddy	*Japonica*	A	–	C	2
JRC 29	Shichimenchou mochi	unknown	upland	*Japonica*	A	–	C	2
JRC 31	Kameji	Shimane	paddy	*Japonica*	A	–	C	2
JRC 32	Omachi	Okayama	paddy	*Japonica*	A	–	C	2
JRC 33	Shinriki	Hyogo	paddy	*Japonica*	A	–	C	2
JRC 34	Kyotoasashi	Kyoto	paddy	*Japonica*	A	–	D	2
JRC 35	Kabashiko	Miyazaki	paddy	*Japonica*	A	–	D	2
JRC 36	Sekiyama	Aomori	paddy	*Japonica*	A	–	C	2
JRC 38	Nagoya shiro	Akita	paddy	*Japonica*	A	–	D	2
JRC 39	Shiroine (KemomiI)	Tokushima	paddy	*Japonica*	A	–	D	2
JRC 40	Akamai	Nagasaki	upland	*Indica*	A	–	D	2
JRC 41	Akamai	Tokushima	paddy	*Indica*	–	348	C	1
JRC 42	Touboshi	Kagoshima	paddy	*Indica*	A	–	D	2
JRC 43	Akamai	Kanto-Tosan region	upland	*Indica*	A	–	D	2
JRC 44	Karahoushi	Kagoshima	paddy	*Indica*	–	348	D	1
JRC 45	Hiyadachitou	Yamagata	paddy	*Japonica*	A	–	C	2
JRC 47	Okabo	unknown	upland	*Japonica*	–	404	C	1
JRC 48	Hakamuri (Yokoyama)	Kagoshima	upland	*Japonica*	–	404	D	1
JRC 49	Rikutou Rikuu 2	unknown	upland	*Japonica*	A	–	C	2
JRC 51	Shinsyuu	Nagano	paddy	*Japonica*	A	–	C	2
JRC 53	Raiden	Kanto-Tosan region	upland	*Japonica*	A	–	C	2
JRC 54	Houmanshinden ine	Kagoshima	paddy	*Japonica*	A	–	C	2
WRC 02	Kasalath	India	paddy	*Indica*	–	348	D	1
WRC 03**	Bei Khe	Cambodia	paddy	*Indica*	–	421	D	1
WRC 04	Jena 035	Nepal	paddy	*Indica*	–	348	C	1
WRC 05**	Naba	India	paddy	*Indica*	–	421	D	1
WRC 06**	Puluik Arang	Indonesia	paddy	*Indica*	–	421	D	1
WRC 07	Davao 1	Philippines	paddy	*Indica*	–	421	D	1
WRC 09**	Ryou Suisan Koumai	China	paddy	*Indica*	–	421	C	1
WRC 10	Shuusoushu	China	paddy	*Indica*	A	–	D	2
WRC 11	Jinguoyin	China	paddy	*Indica*	–	421	D	1
WRC 12**	Dahonggu	China	paddy	*Indica*	–	421	D	1
WRC 13	Asu	Bhutan	paddy	*Indica*	–	421	D	1
WRC 15**	Co 13	India	paddy	*Indica*	–	421	D	1
WRC 16**	Vary Futsi	Madagascar	paddy	*Indica*	–	421	D	1
WRC 17	Keiboba	China	paddy	*Indica*	–	381	C	1
WRC 18	Qingyu (Seiyu)	Taiwan	paddy	*Indica*	–	442	C	1
WRC 20	Tadukan	Philippines	paddy	*Indica*	–	348	D	1
WRC 21**	Shwe Nang Gyi	Myanmar (Burma)	paddy	*Indica*	–	421	C	1
WRC 22	Calotoc	Philippines	paddy	-	–	360	D	1
WRC 23**	Lebed	Philippines	paddy	-	–	421	D	1
WRC 24	Pinulupot 1	Philippines	paddy	-	–	348	C	1
WRC 26	Jhona 2	India	paddy	*Indica*	–	348	C	1
WRC 27*	Nepal 8	Nepal	paddy	*Indica*	–	449	C	1
WRC 28*	Jarjan	Bhutan	paddy	*Indica*	–	449	C	1
WRC 29*	Kalo Dhan	Nepal	paddy	*Indica*	–	449	C	1
WRC 30*	Anjana Dhan	Nepal	paddy	*Indica*	–	449	D	1
WRC 31	Shoni	Bangladesh	paddy	*Indica*	–	348	C	1
WRC 32	Tupa 121-3	Bangladesh	paddy	*Indica*	–	348	C	1
WRC 34	ARC 7291	India	paddy	*Indica*	–	348	C	1
WRC 35	ARC 5955	India	paddy	*Indica*	–	421	C	1
WRC 36	Ratul	India	paddy	*Indica*	–	334	C	1
WRC 37	ARC 7047	India	paddy	*Indica*	–	348	C	1
WRC 38	ARC 11094	India	paddy	*Indica*	–	348	C	1
WRC 39*	Badari Dhan	Nepal	paddy	*Indica*	–	449	C	1
WRC 41	Kaluheenati	Srì Lanka	paddy	*Indica*	–	334	C	1
WRC 44	Basilanon	Philippines	paddy	*Indica*	–	348	C	1
WRC 45	Ma sho	Myanmar (Burma)	paddy	*Tropical Japonica*	–	404	C	1
WRC 46	Khao Nok	Laos	paddy	*Tropical Japonica*	–	360	C	1
WRC 47	Jaguary	Brazil	paddy	*Tropical Japonica*	A	–	C	2
WRC 48	Khau Mac Kho	Vietnam	paddy	*Tropical Japonica*	A	–	C	2
WRC 49	Padi Perak	Indonesia	paddy	*Tropical Japonica*	–	404	C	1
WRC 51	Urasan 1	Japan	upland	*Tropical Japonica*	–	404	C	1
WRC 52	Khau Tan Chiem	Vietnam	paddy	*Tropical Japonica*	A	–	C	2
WRC 53	Tima	Bhutan	paddy	*Tropical Japonica*	A	–	D	2
WRC 58	Neang Menh	Cambodia	paddy	*Indica*	–	348	D	1
WRC 59**	Neang Phtong	Cambodia	paddy	*Indica*	–	433/421	D	1
WRC 60**	Hakphaynhay	Laos	paddy	*Indica*	–	421	C	1
WRC 61	Radin Goi Sesat	Malaysia	paddy	*Indica*	–	421	C	1
WRC 62**	Kemasin	Malaysia	paddy	*Indica*	–	421	C	1
WRC 63	Bleiyo	Thailand	paddy	*Indica*	–	421	C	1
WRC 64	Padi Kuning	Indonesia	paddy	*Indica*	–	421	C	1
WRC 65*	Rambhog	Indonesia	paddy	*Indica*	–	449	C	1
WRC 66	Bingala	Myanmar (Burma)	paddy	*Indica*	–	348	C	1
WRC 67	Phulba	India	paddy	*Japonica*	A	–	C	2
WRC 68	Khao Nam Jen	Laos	paddy	*Japonica*	A	–	C	2
WRC 97	Chin Galay	Myanmar (Burma)	paddy	*Indica*	–	348	C	1
WRC 100	Vandaran	Srì Lanka	paddy	*Indica*	–	421	C	1

A total of 97 (41 Japanese and 56 from the rest of the world) rice landraces were genotyped using Stvb-i-linked markers. Prefix JRC and WRC represent the Japanese collection and world collection, respectively. [a] The ID was obtained from NARO Genebank Project (Tsukuba, Japan). [b] Origin indicates the collection place, area, or prefecture in Japan for JRC and country for WRC landraces. *, **, allelic gene has stop codon in the exon 3. [c] The fragment size amplified by ST5 was measured by fragment analysis using a DNA analyzer ABI 3730xl. A, amplified STrtp-fragment; C, amplified single fragment by ST64; D, amplified multi-fragments by ST64; 1 and 2, genotypes by ST71, single copy and two copies of the 61-bp element, respectively; –, not detected.
